# Supplementary material for: Head and Neck Cancer Patients’ Survival According to HPV Status, miRNA Profiling, and Tumour Features—A Cohort Study
Source: Int J Mol Sci. 2023 Feb 7;24(4):3344. doi: 10.3390/ijms24043344 (PMC9959828; doi:10.3390/ijms24043344)
Supplement: Supplementary file 1 [file ijms-24-03344-s001.zip › ijms-2148635-Supplementary methods - revised V2.pdf]

Supplementary methods for Polymerase Chain Reaction (PCR) HPV testing

Polymerase chain reaction (PCR) for HPV detection was performed with three sets of consensus primers: PGMY, LC and GP5+/GP6+. HPV16 and HPV18 type specific primers were used in multiplex PCR reaction. Beta-globin gene PCR amplification with PC04/GH20 primers was used as internal control of the quality of the isolated DNA and the absence of PCR inhibitors.

Each amplification reaction was carried out in a total volume of 20 µl. The reaction mixtures contained PCR grade water, 1X Green GoTaq® Flexi Buffer (Promega), and 100 ng of each DNA. The conditions and the number of denaturation-annealing extension cycles were different with each set of primers. Primer sequences including original references and expected amplicon sizes are provided in the table below. All PCR experiments contained positive controls including CaSki and/or HeLa cell line DNA and size markers, while the negative PCR controls containing all PCR reagents without DNA.

In the first multiplex reaction, the DNA was amplified with the consensus PGMY primer set and the PC04 and GH20 primers specific for human beta-globin gene. The PCR mixture contained 3 mM $MgCl_2$, 0.1 mM of each dNTP, 0.2 U GoTaq Hot Start DNA Polymerase (Promega), 1.8 µM of each PGMY primer and 50 mM of PC04 and GH20 primers. The DNA amplification was carried out with the following protocol: denaturation at 95°C for 10 min, and 40 cycles which included the denaturation at 95°C for 1 min, the annealing at 55°C for 1 min, the primer extension at 72°C for 1 min, with the final extension at 72°C for 5 min.

The PCR mixture for L1C1/L1C2-1/L1C2-2 consensus primers contained 2.5 mM $MgCl_2$, 0.1 mM of each dNTP, 0.2 U GoTaq DNA Polymerase (Promega), 0.5 µM of L1C1 and 0.25 µM L1C2-1 and L1C2-2 primers. The DNA amplification conditions were: denaturation at 95°C for 10 min, and 30 cycles which included the denaturation at 95°C for 30 s, the annealing at 53°C for 30 s, the primer extension at 72°C for 30s, and the final extension at 72°C for 7 min.

The PCR mixture for GP5+/GP6+ consensus primers contained 2.5 mM $MgCl_2$, 0.05 mM of each dNTP, 0.2 U GoTaq DNA Polymerase (Promega), and 0.5 µM of each primer. The DNA amplification was carried out with the following protocol: denaturation at 95°C for 10 min, and 45 cycles which included the denaturation at 95°C for 1 min, the annealing at 50°C for 2 min, the primer extension at 72°C for 90s, and the final extension at 72°C for 4 min.

The multiplex PCR mixture for HPV16 and HPV18 type specific primers contained 1.625 mM $MgCl_2$, 0.15 mM of each dNTP, 0.2 U GoTaq Hot Start DNA Polymerase (Promega), 0.15 µM of each HPV16 and HPV18 primer. The DNA amplification conditions were: denaturation at 95°C for 10 min, and 35 cycles which included the denaturation at 95°C for 30 s, the annealing at 58°C for 4 min, the primer extension at 72°C for 1 min, with the final extension at 72°C for 7 min.

HPV16 E6 mRNA analysis was performed on HPV 16 DNA positive samples. Briefly, one µg of RNA was reverse transcribed using QuantiTect Reverse Transcription kit (Qiagen) according to the manufacturer's protocol. The presence of full length (~260 bp) or most abundant splice variant (~86bp) of the HPV16 E6 open reading frame (E6*I) was detected. The PCR mixture contained 1.5 mM $MgCl_2$, 0.2 mM of each dNTP, 0.2 U GoTaq Hot Start DNA Polymerase (Promega), 0.25 µM of primer. The DNA amplification conditions were: denaturation at 95°C for 4 min, and 40 cycles which included the denaturation at 95°C for 30 s, the annealing at 55°C for 1 min, the primer extension at 72°C for 90 s, with the final extension at 72°C for 4 min.

Aliquots of each PCR product (10 µl) were analysed by electrophoresis on 2% agarose gels stained with Midori Green Advance dye and visualized by UVItec Cambridge (Alliance 4.7) imaging system.

Table 1. PCR primers used in the study

	Size (bp)	Primer	Sequence	Reference
**β-globin**	268	PC04	5’-GAAGAGCCAAGGACAGGTAC-3’	[34]
		GH20	5’-CAACTTCATCCACGTTCACC-3’	
**PGMY**	~450	PGMY11-A	5’-GCACAGGGACATAACAATGG-3’	[35]
		PGMY11-B	5’-GCGCAGGGCCACAATAATGG-3’	
		PGMY11-C	5’-GCACAGGGACATAATAATGG-3’	
		PGMY11-D	5’-GCCCAGGGCCACAACAATGG-3’	
		PGMY11-E	5’-GCTCAGGGTTTAAACAATGG-3’	
		PGMY09-F	5’-CGTCCCAAAGGAAACTGATC-3’	
		PGMY09-G	5’-CGACCTAAAGGAAACTGATC-3’	
		PGMY09-H	5’-CGTCCAAAAGGAAACTGATC-3’	
		PGMY09-Ia	5’-GCCAAGGGGAAACTGATC-3’	
		PGMY09-J	5’-CGTCCCAAAGGATACTGATC-3’	
		PGMY09-K	5’-CGTCCAAGGGGATACTGATC-3’	
		PGMY09-L	5’-CGACCTAAAGGGAATTGATC-3’	
		PGMY09-M	5’-CGACCTAGTGGAAATTGATC-3’	
		PGMY09-N	5’-CGACCAAGGGGATATTGATC-3’	
		PGMY09-Pa	5’-GCCCAACGGAAACTGATC-3’	
		PGMY09-Q	5’-CGACCCAAGGGAAACTGGTC-3’	
		PGMY09-R	5’-CGTCCTAAAGGAAACTGGTC-3’	
		HMB01b	5’-GCGACCCAATGCAAATTGGT-3’	
**GP5+/6+**	150	GP5+	5’-TTTGTTACTGTGGTAGATACTAC-3’	[36]
		GP6+	5’-GAAAAATAAACTGTAAATCATATTC-3’	
**LC**	250	L1C1	5’-CGTAAACGTTTTCCCTATTTTTTT-3’	[37]
		L1C2-1	5’-TACCCTAAATACTCTGTATTG-3’	
		L1C2-2	5’-TACCCTAAATACCCTATATTG-3’	
**HPV-16**	253	F HPV-16	5’-CCCAGCTGTAATCATGCATGGAGA-3’	[38]
		R HPV-16	5’-GTGTGCCCATTAACAGGTCTTCCA-3’	
**HPV 18**	201	F HPV-18	5’-CGA CAG GAA CGA CTC CAA CGA-3’	[38]
		R HPV-18	5’-GCTGGTAAATGTTGATGATTAACT-3’	
**E6*I**	~260/~86	F HPV16 E6	5’-TTACTGCGACGTGAGGTGTA-3’	[39]
		R HPV16 E6	5’-GGAATCTTTGCTTTTTGTCC-3’	
