# Supplementary material for: Head and Neck Cancer Patients’ Survival According to HPV Status, miRNA Profiling, and Tumour Features—A Cohort Study
Source: Int J Mol Sci. 2023 Feb 7;24(4):3344. doi: 10.3390/ijms24043344 (PMC9959828; doi:10.3390/ijms24043344)

## Slide 1
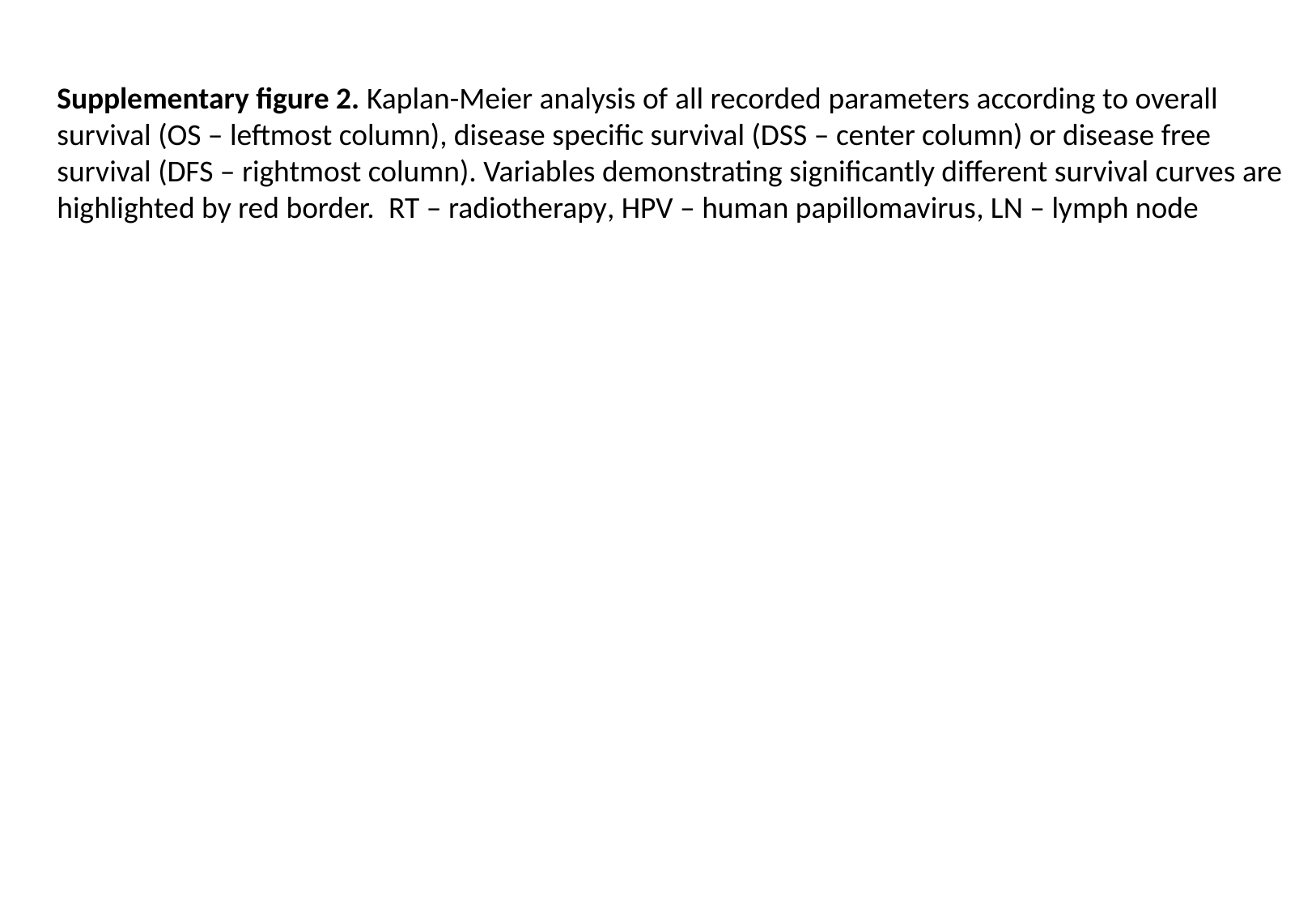

Supplementary figure 2. Kaplan-Meier analysis of all recorded parameters according to overall survival (OS – leftmost column), disease specific survival (DSS – center column) or disease free survival (DFS – rightmost column). Variables demonstrating significantly different survival curves are highlighted by red border. RT – radiotherapy, HPV – human papillomavirus, LN – lymph node

## Slide 2
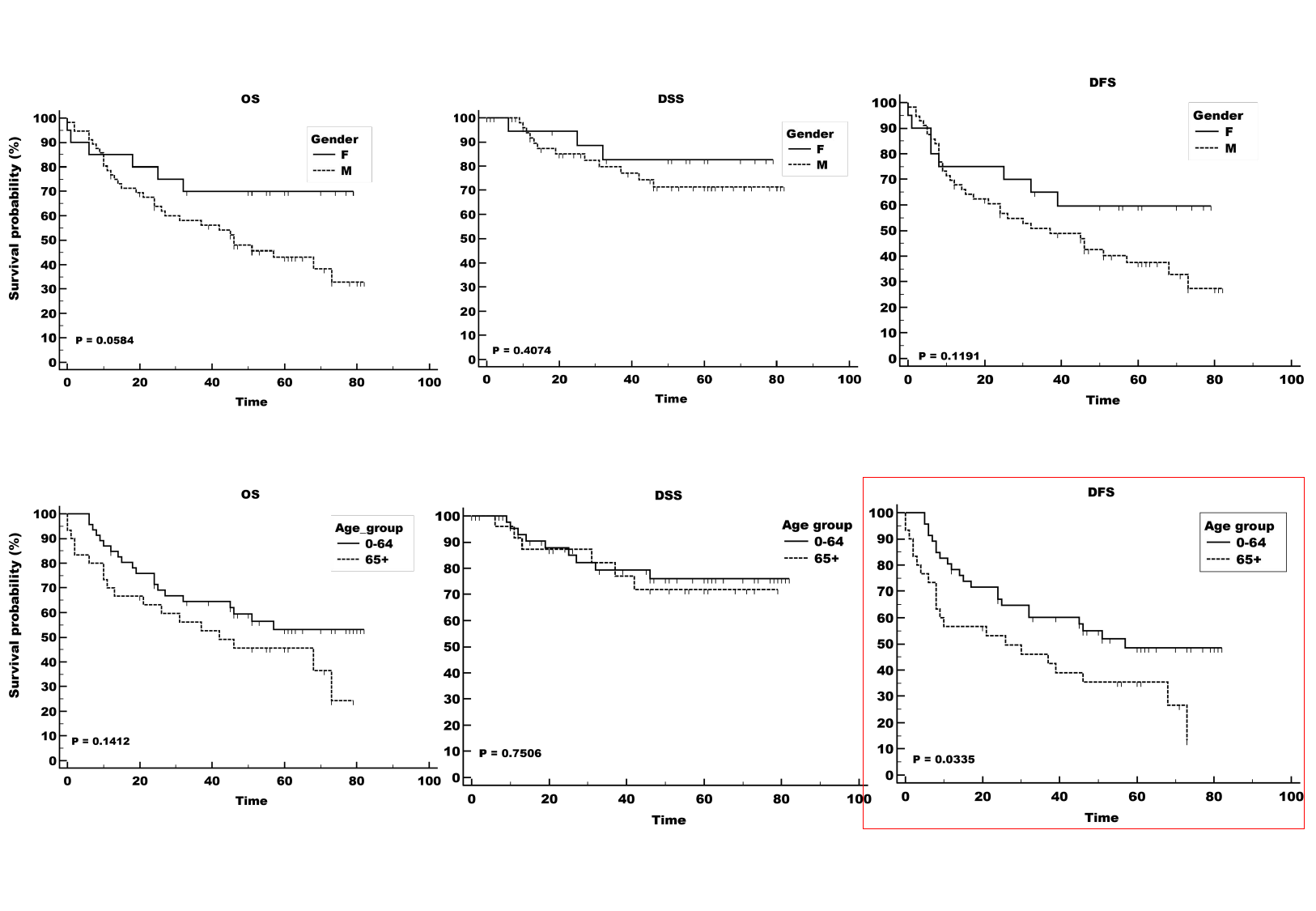

## Slide 3
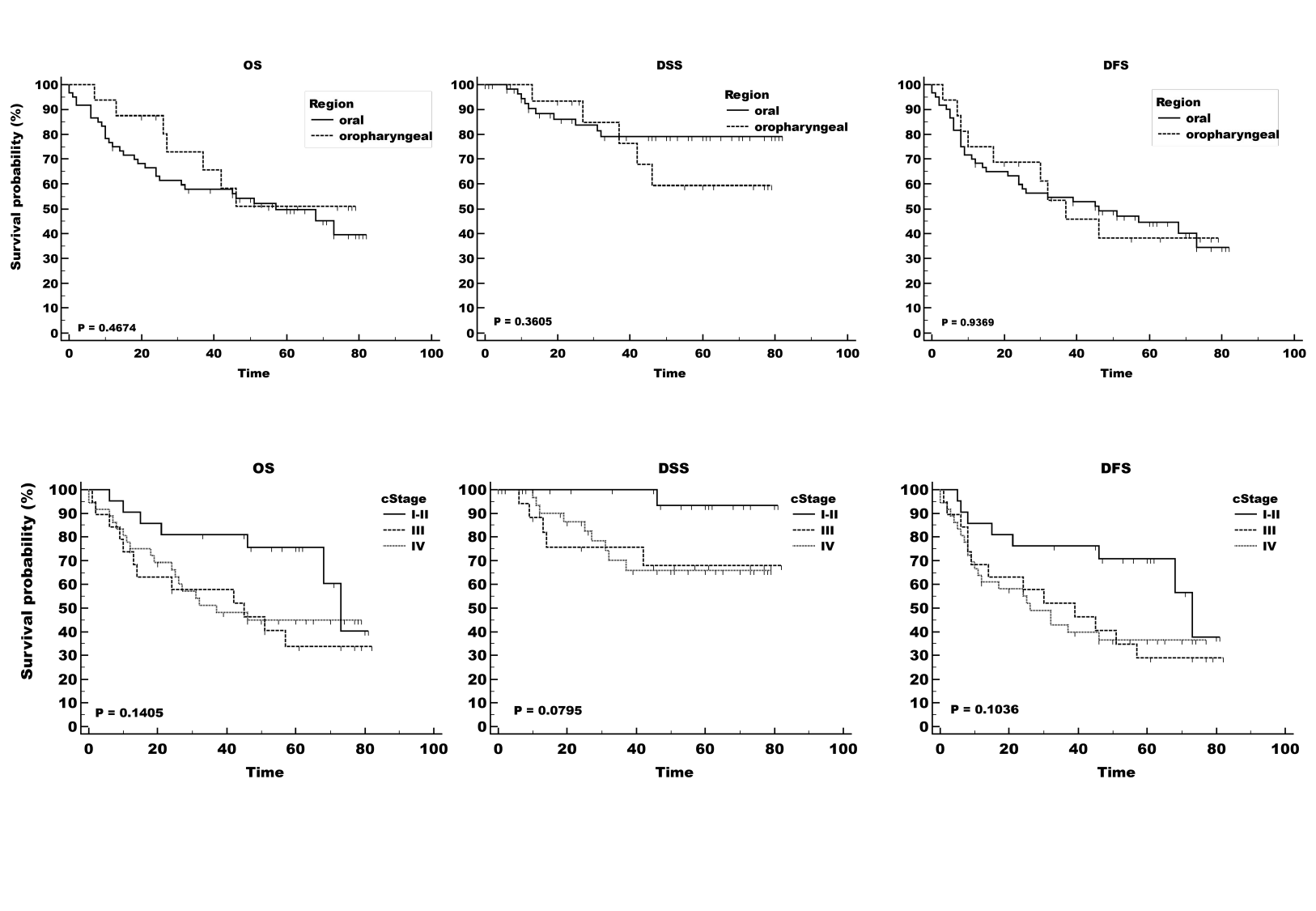

## Slide 4
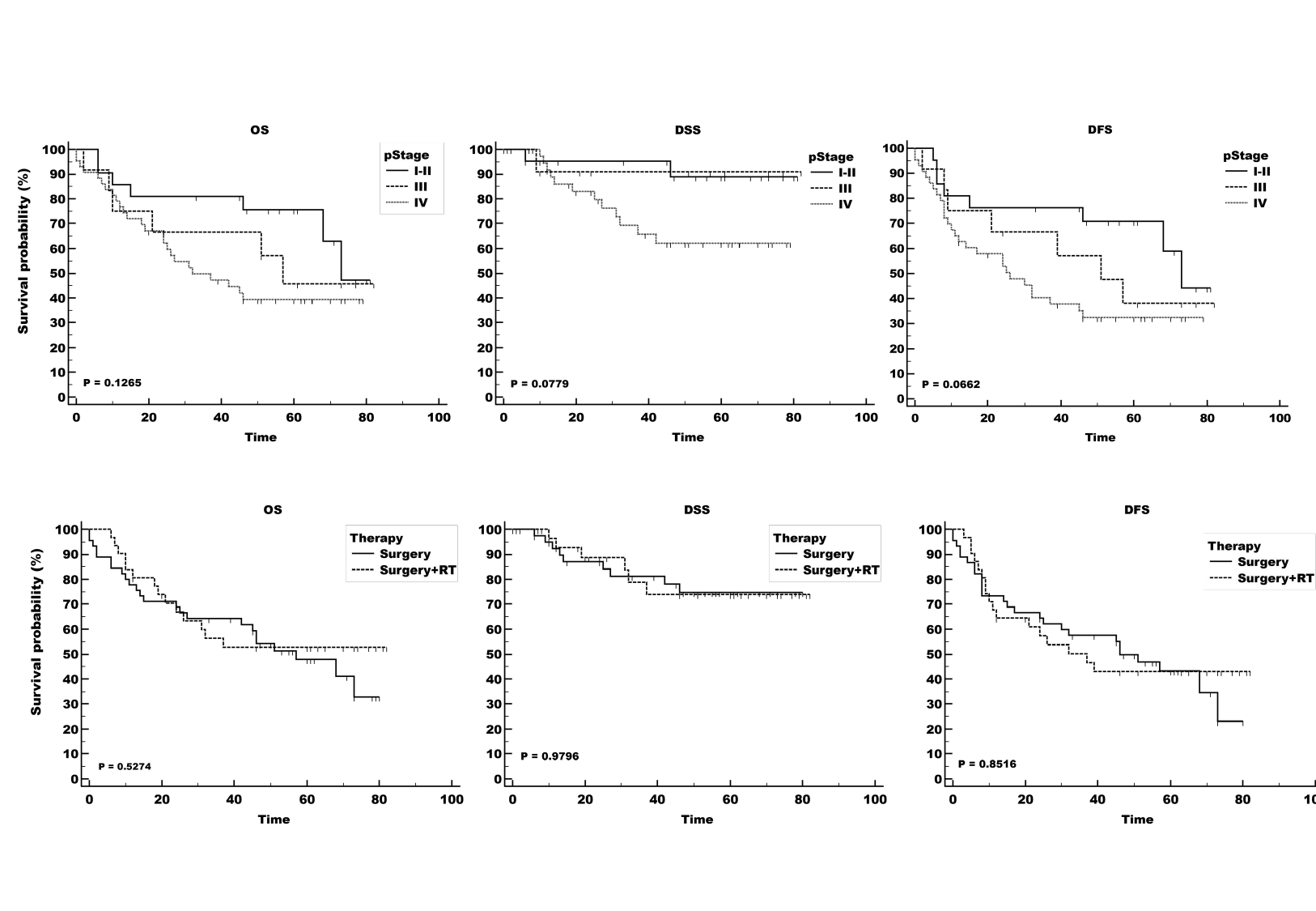

## Slide 5
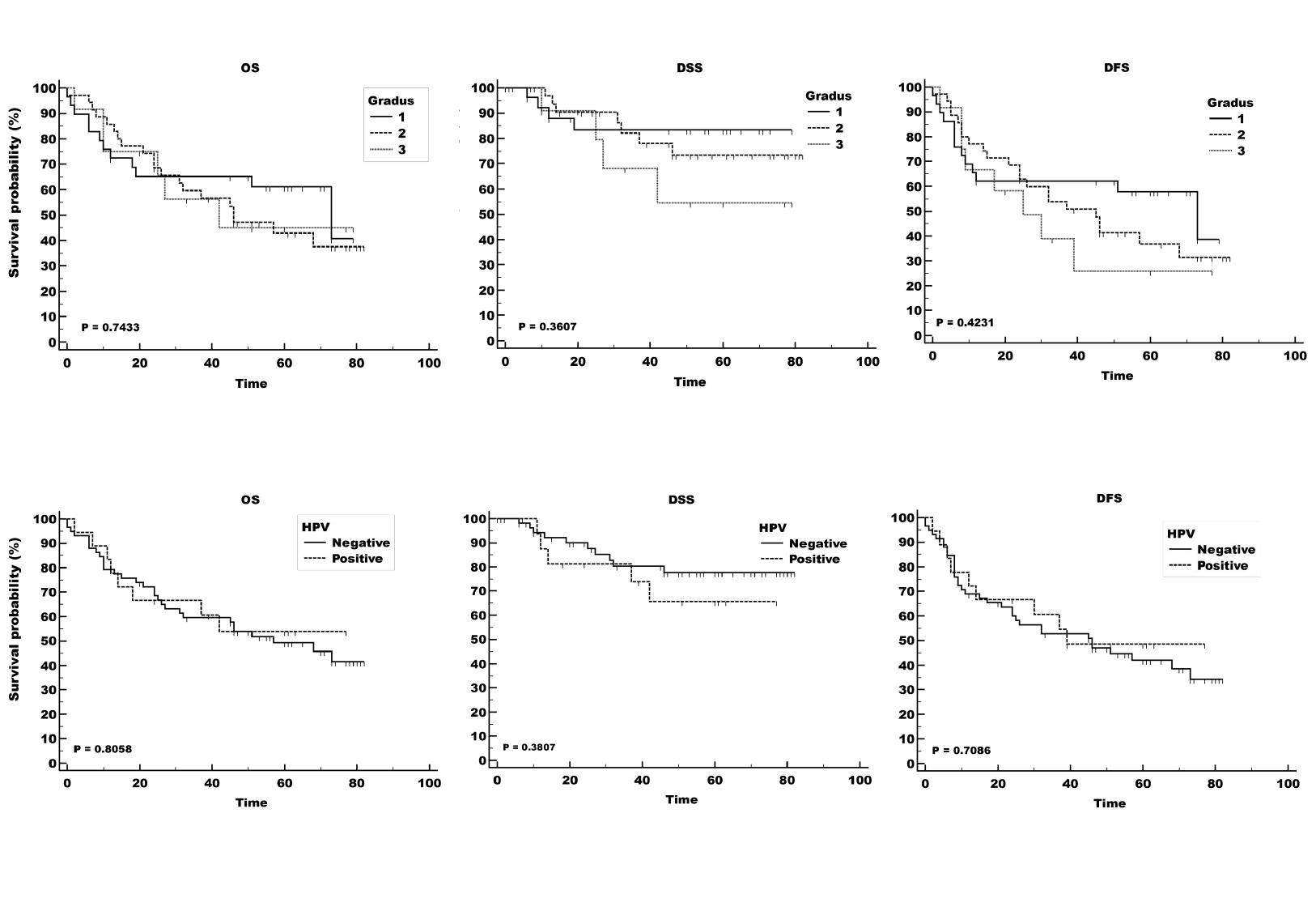

## Slide 6
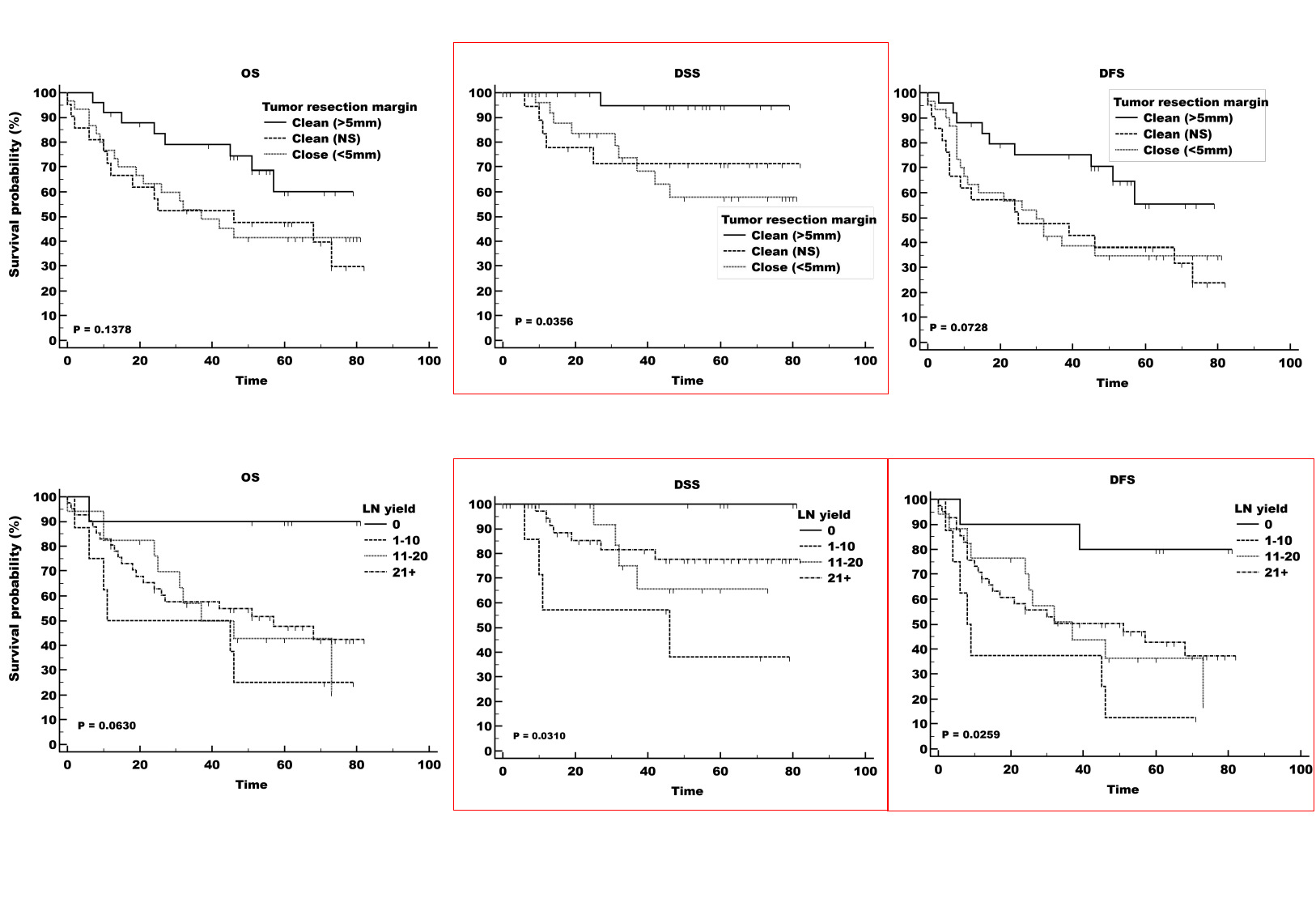

## Slide 7
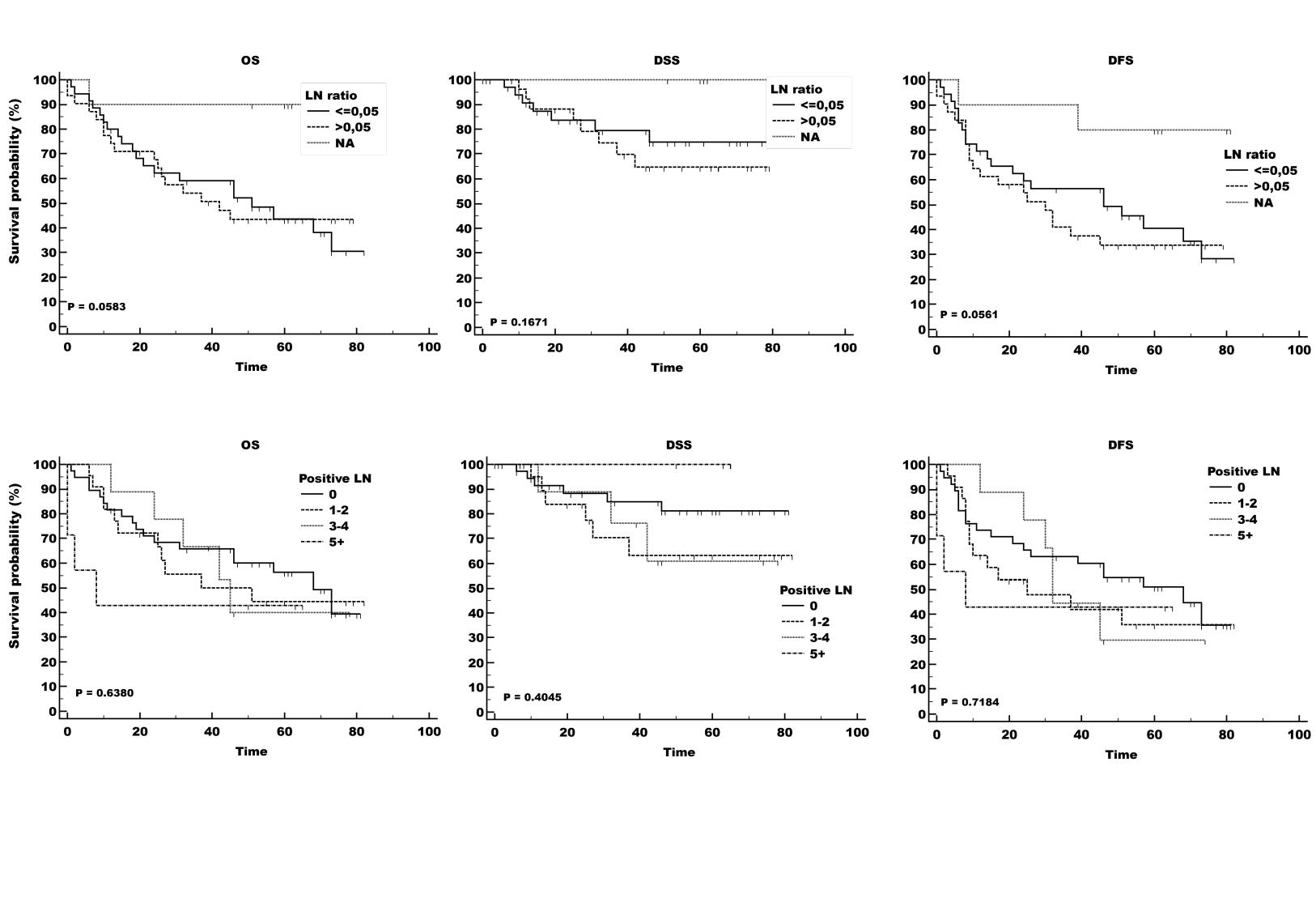

## Slide 8
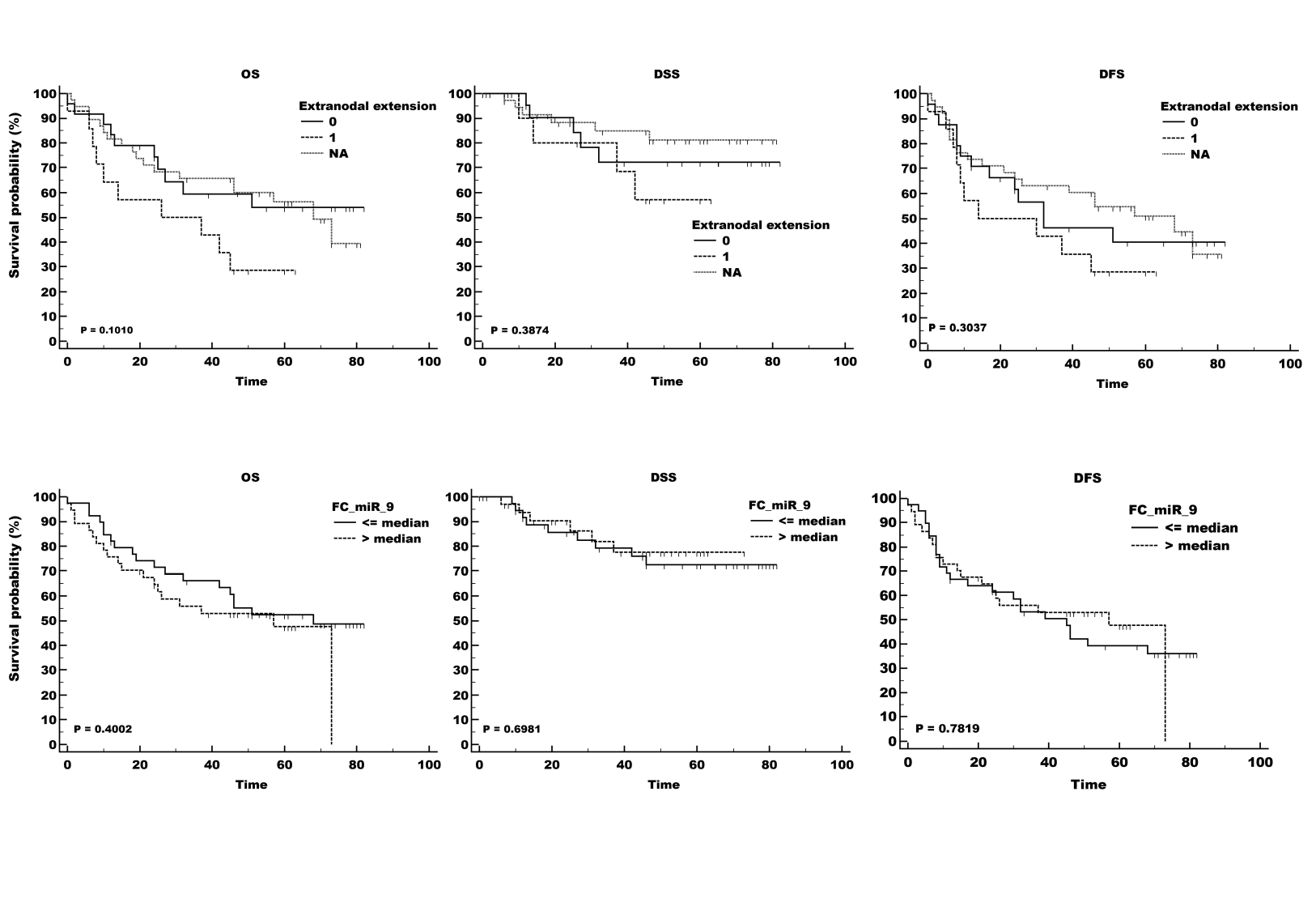

## Slide 9
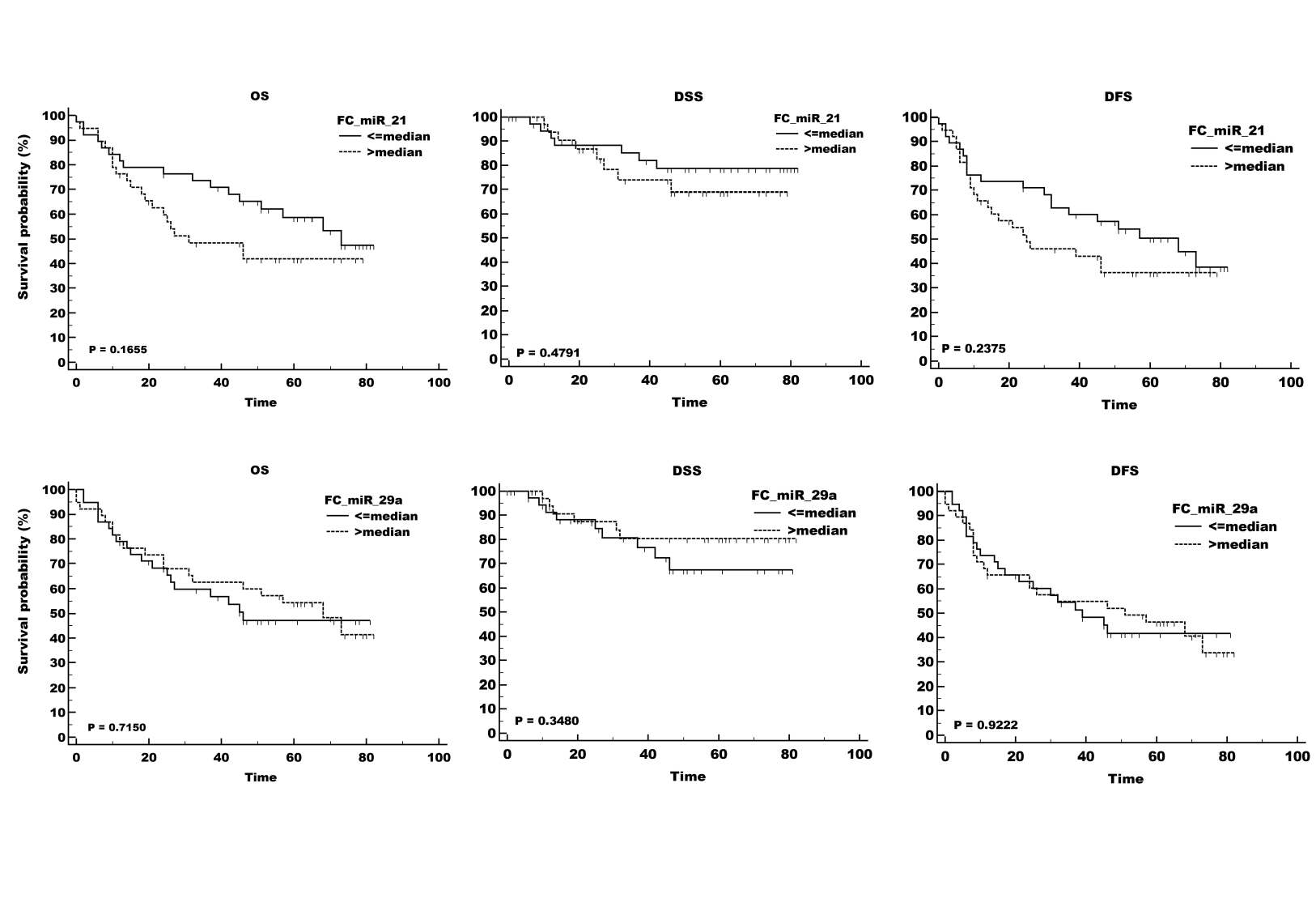

## Slide 10
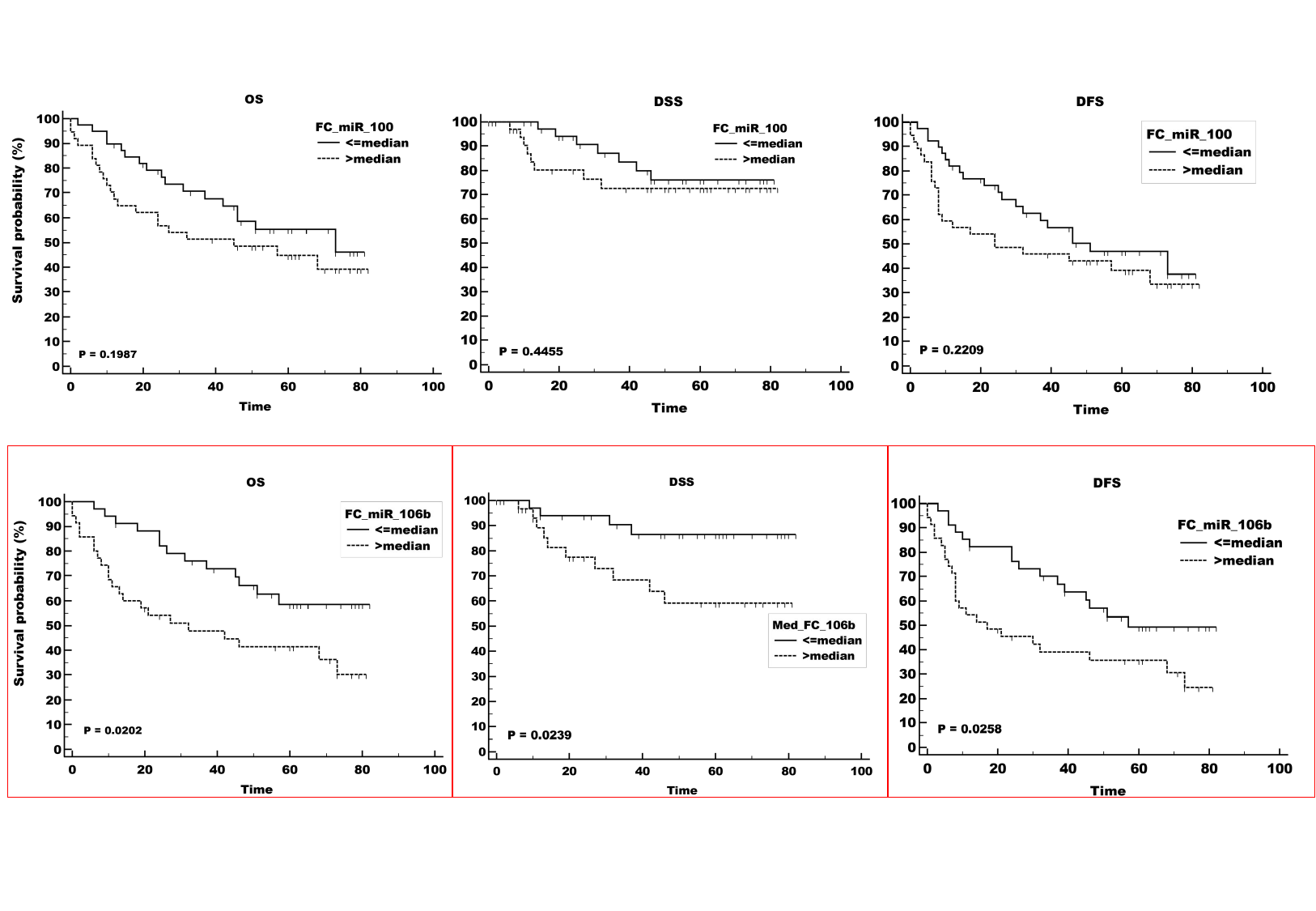

Supplement: Supplementary file 1 [file ijms-24-03344-s001.zip › Supplementary figure 2.pptx]
