# Supplementary material for: Head and Neck Cancer Patients’ Survival According to HPV Status, miRNA Profiling, and Tumour Features—A Cohort Study
Source: Int J Mol Sci. 2023 Feb 7;24(4):3344. doi: 10.3390/ijms24043344 (PMC9959828; doi:10.3390/ijms24043344)

## Slide 1
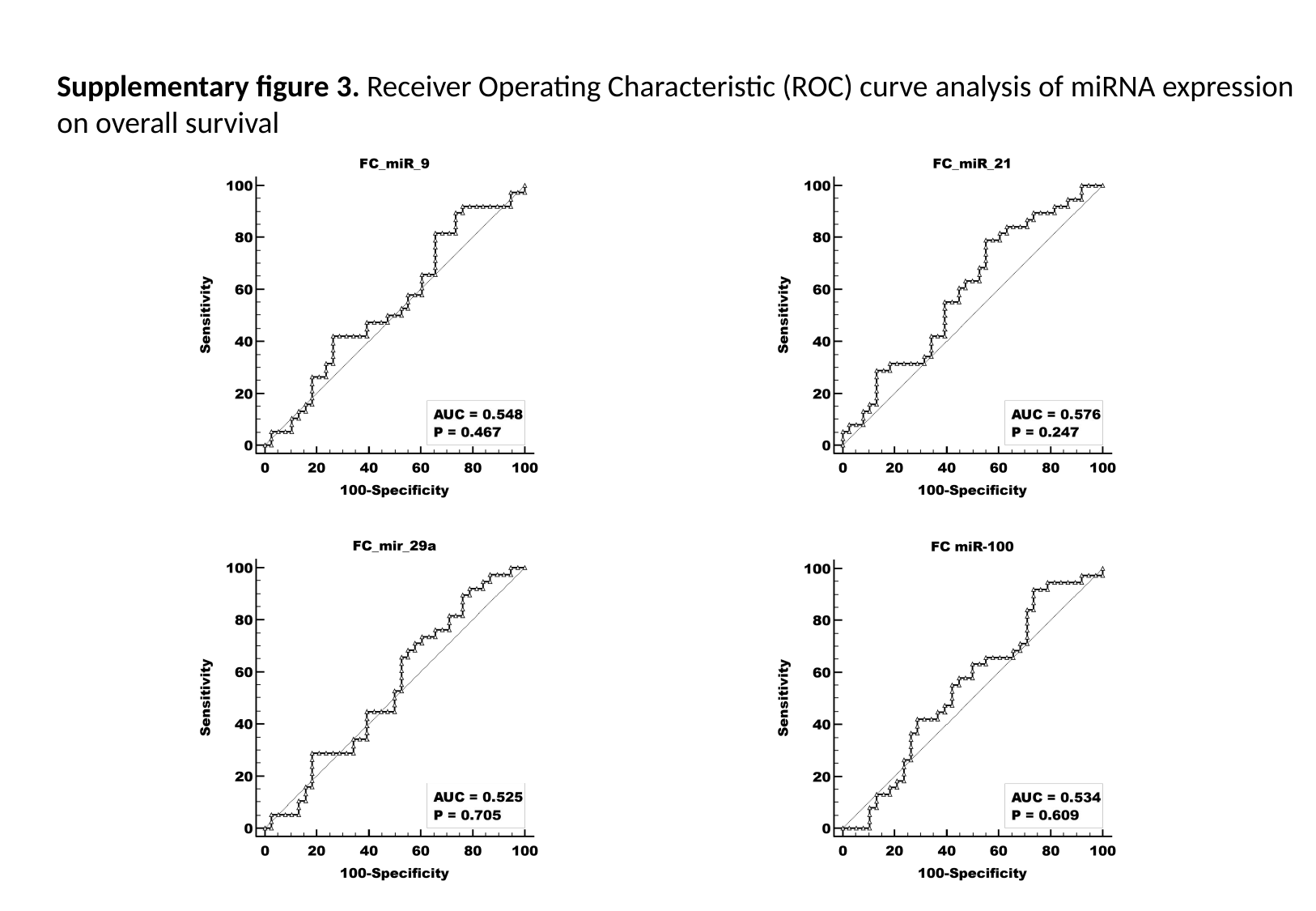

Supplementary figure 3. Receiver Operating Characteristic (ROC) curve analysis of miRNA expression on overall survival

## Slide 2
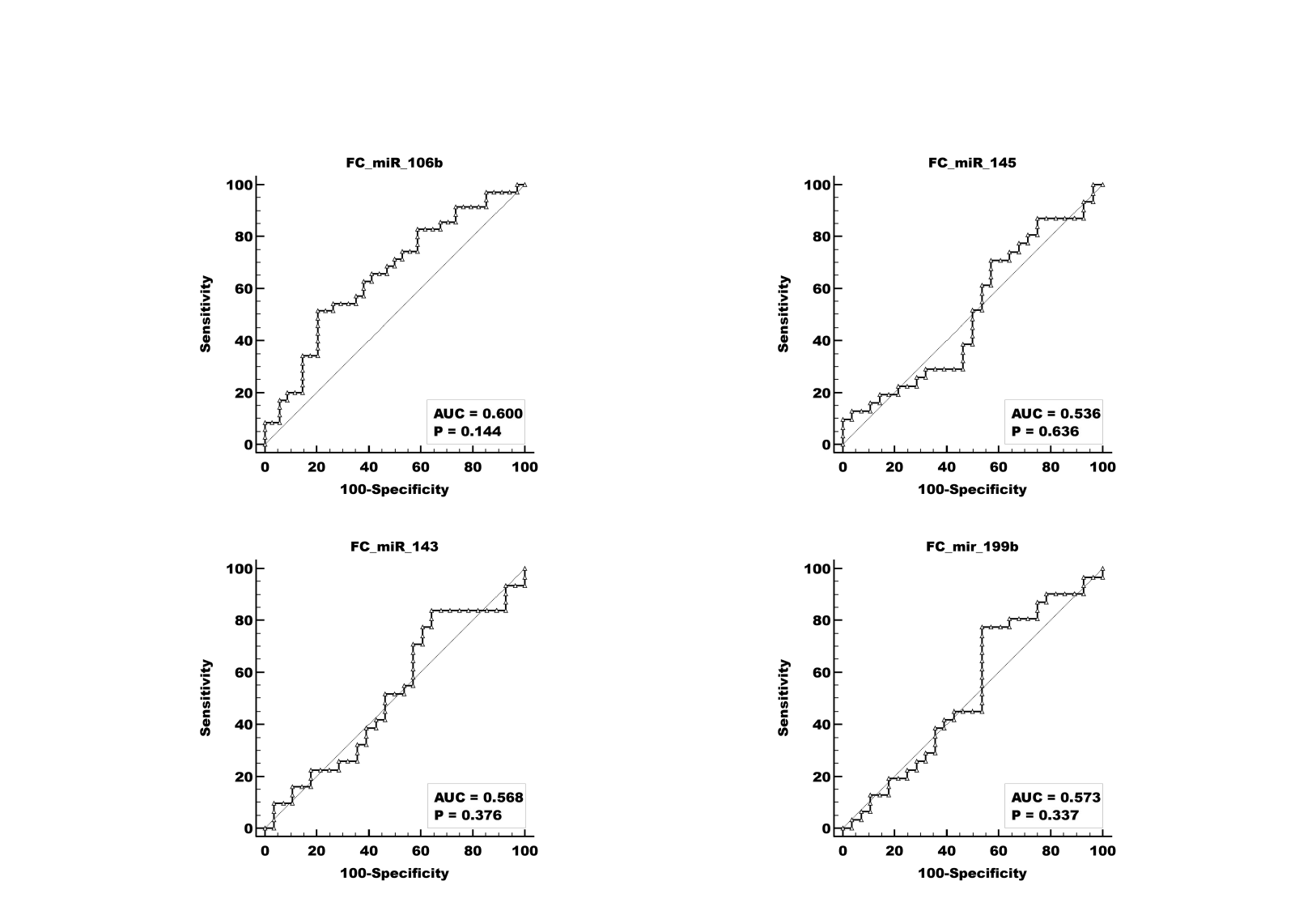

Supplement: Supplementary file 1 [file ijms-24-03344-s001.zip › Supplementary figure 3.pptx]
